# Comorbid Diagnosis of Psychotic Disorders in Borderline Personality Disorder: Prevalence and Influence on Outcome

**DOI:** 10.3389/fpsyt.2018.00084

**Published:** 2018-03-14

**Authors:** C. W. Slotema, Jan D. Blom, Marieke B. A. Niemantsverdriet, Mathijs Deen, Iris E. C. Sommer

**Affiliations:** ^1^Parnassia Psychiatric Institute, The Hague, Netherlands; ^2^Faculty of Social and Behavioural Sciences, Leiden University, Leiden, Netherlands; ^3^Department of Psychiatry, University of Groningen, Groningen, Netherlands; ^4^Department of Neuroscience, University Medical Center Groningen, Groningen, Netherlands; ^5^Department of Psychiatry, University Medical Center Utrecht, Utrecht, Netherlands

**Keywords:** comorbidity, outcome, predictive value, prevalence, psychotic disorder

## Abstract

**Background:**

A diagnosis of psychotic disorder is traditionally considered incompatible with borderline personality disorder (BPD), even though patients sometimes fulfill the diagnostic criteria for both disorders. How often this happens is barely known, as is the influence of comorbid psychotic disorders on the outcome of BPD. Since studies on isolated auditory verbal hallucinations in patients with BPD indicate that these perceptual symptoms have severe consequences and are associated with suicidal behavior and hospitalization, patients with comorbid psychotic disorders are unlikely to fare better.

**Objective:**

To examine the point prevalence of psychotic disorders in patients with BPD, their association with the outcome of BPD, and their predictive value for outcome.

**Methods:**

In a cross-sectional design, 84 female outpatients diagnosed with BPD were interviewed with the aid of the MINI-International Neuropsychiatric Interview to establish the point prevalence of comorbid psychotic and other comorbid disorders. After termination of their treatment at a specialized outpatient clinic, the type of referral was considered to be a “good” outcome when they were referred to their general practitioner or to basic psychiatric care for noncomplex patients, and a “poor” outcome when referred to a specialized psychiatric department or to a psychiatric district team for patients with severe psychiatric disorders.

**Results:**

Psychotic disorders were present in 38% of the patients with BPD. With a prevalence of 20%, psychotic disorder not otherwise specified (NOS) was the most common subtype; the least common types were schizophrenia (2%), substance-induced psychotic disorder (2%), and brief psychotic disorder (1%). Among six types of comorbid disorders, only psychotic disorders were associated with a poor outcome; they were also *predictors* for a poor outcome, along with comorbid mood disorders, eating disorders, and somatoform disorders, as well as the severity of BPD, and, counterintuitively, more years of education.

**Conclusion:**

Psychotic disorders, notably of the psychotic disorder NOS subtype, are common among patients with BPD, and their presence is associated with a poor outcome. This implies that adequate diagnosis and treatment of both disorders is warranted in this subgroup with a dual diagnosis.

## Introduction

Borderline personality disorder (BPD) is conceptualized as a combination of affective dysregulation, impulsive/behavioral dyscontrol, disturbed interpersonal relatedness, and cognitive-perceptual symptoms (1–3). Symptoms of the latter include suspiciousness, ideas of reference, paranoid ideation, illusions, derealization, depersonalization, and hallucination-like symptoms. One of the criteria for BPD is that “transient, stress-related paranoid ideation may be present” (4). Moreover, psychotic features among patients with BPD are “by definition considered as mild and transient and hallucination-like in nature.” BPD is often accompanied by dissociation, intense episodes of anger, and suicidal ideation and behavior, resulting in functional impairment and increased health care utilization (2, 3, 5). Clinical studies indicate that BPD can also be accompanied by comorbid disorders such as mood and anxiety disorders, substance abuse disorders, and eating disorders (6–8). However, few data are available on the prevalence of psychotic disorders in patients with BPD. This is at least partly due to the fact that classifications such as the *Diagnostic and Statistical Manual of Mental Disorders* [DSM-5 (4)] and the *International Classification of Diseases and Related Health Problems* [ICD-10 (9)] stipulate that the presence of a major psychotic disorder precludes BPD as a diagnosis (10). This is unfortunate, as these disorders are characterized by different sets of symptoms and, moreover, hallucinations are experienced by 26–54% of patients diagnosed with BPD (11–14) and delusions by 17–29% (12, 13, 15). The additional burden caused by such psychotic symptoms is often substantial, as is demonstrated, for example, by increased levels of distress, more frequent suicide plans and attempts, and substantially higher needs for hospitalization in patients with BPD who also suffer from auditory verbal hallucinations (16).

Because so little is known about the co-occurrence of psychotic disorders and BPD, it is also unknown to what extent they determine the long-term outcome of BPD, which in itself can vary substantially, allowing patients to either live meaningful and fairly stable lives (whether or not with a partner, children, and a paid job) or end up in a secluded nursing ward with highly specialized care for the remainder of their lives. The long-term outcome of BPD has been investigated in two large-scale prospective cohort studies, i.e., the *Collaborative Longitudinal Personality Disorders Study* (17) and the *McLean Study of Adult Development* (18). In these studies, remission rates ranged from 30 to 50% by the second year of follow-up, to up to 80% by the tenth year, with the mean number of symptoms decreasing steadily over time. Despite these encouraging results, some 20% of the patients under study failed to recover substantially within 10 years. What distinguishes the latter patients from those who did recover is not fully known, even though many variables were found to affect the symptomatic outcome of BPD, including sociodemographic factors, severity of symptoms, personality traits, comorbid posttraumatic stress disorder (PTSD), and comorbid substance abuse disorders, whereas the co-occurrence of mood disorders, anxiety disorders, and eating disorders did not negatively affect outcome (19–21). However, since comorbid psychotic disorders, such as schizophrenia, were an exclusion criterium in the above-mentioned studies, their influence on the long-term outcome of BPD was not assessed.

As insight into the predictive factors for the outcome of BPD is urgently needed to allow for optimization of treatment and prevention of chronic hospitalization, the present study was designed to answer the following questions:

1. What is the point prevalence of comorbid psychotic disorders in patients diagnosed with BPD?
2. Is the long-term outcome of BPD associated with the presence of comorbid psychotic disorders?
3. Can individual disorders or conditions be identified that predict outcome in patients with BPD?

## Materials and Methods

### Participants

Patients with BPD, diagnosed in accordance with the criteria issued by the DSM-IV-TR (1) between May 2012 and March 2015, were recruited at the Department of Personality Disorders (an outpatient clinic specialized in the treatment of personality disorders) at Parnassia Psychiatric Institute, The Hague. Data collection for this study was part of a treatment protocol described in Niemantsverdriet et al. (14). All patients referred to the clinic were help seeking. The majority of patients were referred by their primary clinician and the remainder by other departments of Parnassia Psychiatric Institute. Criteria for inclusion were (i) a diagnosis of BPD as established with the aid of the Structural Clinical Interview for Axis II Personality Disorders (22), (ii) age ≥18 years, and (iii) written, informed consent. The study was approved by Parnassia’s Institutional Review Board (registration number 6237) in accordance with the Declaration of Helsinki.

### Instruments and Procedure

For this cross-sectional study, the presence of psychotic disorders and other comorbid disorders was assessed with the aid of the MINI-International Neuropsychiatric Interview [MINI PLUS (23)]. Thus, the presence of seven groups of disorders *at that moment* was explored, comprising mood disorders (i.e., depressive disorder, bipolar disorder), anxiety disorders (i.e., panic disorder, agoraphobia, social phobia, obsessive-compulsive disorder, and general anxiety disorder), substance abuse disorders (i.e., alcohol dependence, alcohol abuse, substance (non-alcohol) abuse, and substance dependence), PTSD, eating disorders (i.e., anorexia nervosa and bulimia nervosa), somatoform disorders (i.e., hypochondriasis, body dysmorphic disorder, and pain disorder), and psychotic disorders (i.e., schizophrenia, schizoaffective disorder, schizophreniform disorder, brief psychotic disorder, delusional disorder, psychotic disorder due to a general medical condition, substance-induced psychotic disorder, psychotic disorder not otherwise specified (NOS), major depressive disorder with psychotic features, and bipolar I disorder with psychotic features). Thus, we defined psychotic disorders as disorders listed in the DSM-5 under the heading of Schizophrenia Spectrum and Other Psychotic Disorders, plus three diagnostic categories that were included in the MINI PLUS, i.e., psychotic disorders due to a medical condition, mood disorders with psychotic features, and substance abuse disorders with psychotic features. Kappa coefficient, sensitivity, and specificity were good or very good for all diagnoses (i.e., kappa coefficients varied from 0.76 to 0.93), with the exception of those for generalized anxiety disorder (kappa = 0.36), agoraphobia (sensitivity = 0.59), and bulimia nervosa (kappa = 0.53 (23)). Inter-rater and test-retest reliability were good.

Interviewers were two psychologists, two residents of psychology, and one resident of psychiatry who had been trained in conducting the MINI PLUS. They participated in monthly meetings to safeguard the inter-rater reliability during the inclusion phase.

At the outpatient clinic, patients generally remained under treatment as long as specialized psychiatric care was expected to enable further improvement of their BPD symptoms. When this could no longer be reasonably expected, each patient was offered four options for further care, comprising (i) referral to their general practitioner when psychiatric care was no longer necessary, (ii) referral to basic psychiatric care for noncomplex patients when they were expected to need ≤800 min of care per year, and crisis management was no longer necessary, (iii) referral to another specialized psychiatric department when treatment of a comorbid disorder was necessary, and (iv) referral to a psychiatric district team for patients with severe psychiatric disorders when the estimated duration of further treatment was ≥2 years, problems in psychosocial functioning were present, and adjuvant social care was needed. For the present study, outcomes (i) and (ii) were defined as “good” and outcomes (iii) and (iv) as “poor.” After termination of care at the outpatient clinic, the type of follow-up treatment for each individual patient could be retrieved from their electronic medical records.

### Statistical Analyses

Data analysis was performed with SPSS version 23 and R version 3.3.2. Demographic differences between the two groups were analyzed with Chi^2^ tests, *t*-tests for independent samples, and the Mann–Whitney *U*-test, as the duration of treatment was not normally distributed. Differences in outcome between the groups (i.e., good versus poor outcome) in relation to comorbid disorders were analyzed with Chi^2^ tests and odds ratios (OR). The Benjamini–Hochberg correction was used to correct for multiple testing (24). To search for predictive factors for good and poor outcome, a logistic regression analysis was performed. As the number of patients was relatively small, we applied elastic net regularization (25) using the glmnet package (26) within R (27). After exclusion of all cases with missing data, the dataset was changed into a dataset with all predictors, and a vector Y with the outcome variables. A regression analysis was conducted by penalizing regression coefficients to 0, i.e., by decreasing the variance of the predictors toward 0. Whenever a variable had regression coefficient 0, it was erased from the regression model. Two-dimensional cross-validation was used to explore which combination of penalization degree and mixture led to the smallest prediction error (28) applying the one-standard-error rule (29). The dependent value was outcome (i.e., good versus poor), whereas independent values were age, total years of education, total number of BPD criteria, and comorbid disorders.

## Results

### Descriptives

Included were 84 female patients (and no male patients) with BPD; their demographic data are presented in Table 1. The treatment of all patients consisted of psychodynamic psychotherapy, cognitive-behavioral therapy, schema-focused therapy or dialectical behavior therapy, and supporting sessions, either individually or in a group. The choice of treatment depended on the patient’s capacity to participate within a group setting, their introspective ability, and the education of the therapist.

**Table 1:** Demographic data of patients with borderline personality disorder (BPD) with a good and bad long-term outcome.

Demographic variables	Good outcome (*n* = 40)	Poor outcome (*n* = 44)	Analysis	*p*-Value
Age in years (mean, SD)	36 (11.5)	38.5 (12)	*t* = 0.985	0.33
Years of education (mean, SD)	11.1 (2.4)	12.2 (2.4)	*t* = 1.924	0.058
Duration of treatment in months (median)	65	70	MWUT	0.38
Number of BPD criteria (mean, SD)	6.4 (1.4)	6.7 (1.4)	*t* = 1.073	0.29
**Medication**				
Antipsychotics (%, number)	34% (13)	41% (18)	FET	0.50
Antidepressants (%, number)	38% (15)	55% (23)	FET	0.19
Mood stabilizers (%, number)	8% (3)	5% (2)	FET	0.66
Benzodiazepines (%, number)	24% (9)	31% (13)	FET	0.62
Total number of psychopharmacological agents (mean, SD)	1.1 (1.1)	1.4 (1.1)	*t* = 1.291	0.20

*FET, Fisher’s Exact test; MWUT, Mann–Whitney U-test*.

In addition, many of the patients received medication, with half of those with a comorbid psychotic disorders using antipsychotics. Treatment duration ranged from 10 to 192 months, with 89% of the patients being under treatment for ≥24 months.

### Comorbidity

The point prevalence of comorbid disorders was 17–70%, with anxiety disorders being the most prevalent ones and eating disorders the least prevalent ones (Table 2). Psychotic disorders were present in 38% of the patients. With a point prevalence of 20%, psychotic disorder NOS was the most common of these, whereas schizophreniform disorder and mood disorders with psychotic features were present in 6 and 7% of the patients, respectively. Two patients (2%) fulfilled the criteria of schizophrenia and three (4%) the criteria of schizoaffective disorder.

**Table 2:** Frequency of comorbid disorders present in at least one patient with borderline personality disorder (total group = 84 patients).

Comorbid disorder	% (number)
Mood disorder	54 (45)
Anxiety disorder	70 (59)
Posttraumatic stress disorder	41 (34)
Substance abuse disorder	32 (27)
Eating disorder	17 (14)
Somatoform disorder	36 (30)
Psychotic disorders	38 (31)
Mood disorder with psychotic features	6 (5)
Schizophrenia	2 (2)
Schizoaffective disorder	4 (3)
Brief psychotic disorder	1 (1)
Substance-induced psychotic disorder	2 (2)
Psychotic disorder not otherwise specified	20 (17)
Bipolar I disorder with psychotic features	1 (1)

### Outcome

Of all 84 patients, 40 (48%) were identified as having a good outcome and 44 (52%) as having a poor outcome. No statistical difference was found between these two groups regarding demographic factors at baseline, use of medication, or duration of treatment. Table 3 shows the associations between all comorbid disorders and outcome. The association between psychotic disorders and outcome was significant (OR 3.0; 95% CI 1.175–7.657; *p* = 0.024) but was not significant for all other comorbid disorders.

**Table 3:** Associations between outcome and comorbid disorders in at least one patient with borderline personality disorder (total group = 84 patients).

Disorder, % (number)	Good outcome (*n* = 40)	Poor outcome (*n* = 44)	*p*-Value	Odds ratios	95% CI
Mood disorder	48% (19)	59% (26)	0.38	1.6	0.673–3.787
Anxiety disorder	65% (26)	75% (33)	0.35	1.6	0.630–4.145
Posttraumatic stress disorder	35% (14)	46% (20)	0.38	1.5	0.642–3.731
Substance abuse	28% (11)	36% (16)	0.48	1.5	0.596–3.806
Eating disorder	13% (5)	21% (9)	0.39	1.8	0.548–5.913
Somatoform disorder	30% (12)	41% (18)	0.36	1.6	0.654–3.992
Psychotic disorders	25% (10)	50% (21)	0.024	3.0	1.175–7.657
Mood disorder with psychotic features	3% (1)	9% (4)	0.36	3.9	0.417–36.461
Schizophrenia	0	5% (2)	0.50	–	–
Schizoaffective disorder	0	7% (3)	0.24	–	–
Brief psychotic disorder	3% (1)	0	0.48	–	–
Substance-induced psychotic disorder	3% (1)	2% (1)	1.0	0.93	0.056–15.360
Psychotic disorder not otherwise specified	18% (7)	23% (10)	0.80	1.2	0.413–3.354
Bipolar I disorder with psychotic features	0	2% (1)	1.0	–	–

### Predicting Outcome

Regarding the logistic regression analysis, the variables that were preserved in the model with optimal penalization were mood disorders, psychotic disorders, eating disorders, somatoform disorders, more years of education, and total number of BPD criteria with the highest OR for eating disorders (Table 4).

**Table 4:** Logistic regression analysis for age, total years of education, total number of borderline personality disorder (BPD) criteria, comorbid disorders, and outcome in patients with BPD.

Factor	Regression coefficients	Odds ratios
Years of education	−0.139	0.87
Total number of BPD criteria	−0.0769	0.93
Mood disorders	−0.0366	0.96
Psychotic disorders	−0.108	0.90
Eating disorders	−0.577	0.56
Somatoform disorders	−0.357	0.70

## Discussion

This study investigated the point prevalence of psychotic disorders in 84 female patients diagnosed with BPD and the association of these disorders with outcome. In addition, other comorbid disorders, demographic variables, and the severity of BPD were explored as predictive factors for outcome. All seven groups of comorbid disorders that we assessed were represented in our sample, with prevalence rates ranging from 17% for eating disorders to 70% for anxiety disorders. Psychotic disorders were present in 38% of the patients, with psychotic disorder NOS as the most common variant (20%). Schizophrenia was present in 2% of the patients and schizoaffective disorder in 4%. Despite the high prevalence rate of mood disorders among patients with BPD (93%), mood symptoms did rarely contribute to a diagnosis of psychotic disorders. A significant correlation was found between psychotic disorders and (poor) outcome. Psychotic disorders also constituted a *risk factor* for poor outcome, along with mood disorders, eating disorders, and somatoform disorders, as well as the severity of BPD, and more years of education.

The relatively small numbers of patients diagnosed with schizophrenia and schizoaffective disorder are in accordance with the studies of Thompson et al. (30), Kingdon et al. (12), and Turner et al. (31). The remaining diagnostic categories of the group of psychotic disorders have hardly been studied in patients with BPD. In a prior study of 379 inpatients with BPD, 1.3% had a lifetime diagnosis of psychotic disorders, as established with the aid of the Structured Clinical Interview for DSM-III-R (7), with no further distinction being made for the specific type of psychotic disorder. Thompson et al. (30) explored the transition to psychosis within 24 months in 14 outpatients with an ultra-high risk for psychosis and BPD; of whom, 13% developed a schizophrenia spectrum disorder (i.e., schizophrenia, schizoaffective disorder, or schizophreniform disorder) and 10% “other psychotic disorders” (i.e., brief psychotic disorder, delusional disorder, psychotic disorder due to a medical condition, substance-induced psychotic disorder, or substance-induced mood disorder with manic features and psychotic disorder NOS); no differences in prevalence rates between these groups were reported. In a community population of 46 patients with non-suicidal self-injury and BPD, the lifetime prevalence of psychotic disorders was 4.5% (31). More specifically, schizoaffective disorder, substance-induced psychotic disorder, and psychotic disorder NOS were present in 2.3, 2.3, and 9.5% of the patients, respectively. Finally, in a study by Kingdon et al. (12) among 52 inpatients and outpatients with BPD, 37% had BPD as well as schizophrenia; the presence of other types of schizophrenia spectrum disorder was not investigated. “Exploring the presence of BPD in patients with schizophrenia reveals more or less similar prevalence rates, i.e., 2 to 7% (32–35).”

The present study is the first to report on the point prevalence of all types of psychotic disorders in conjunction with BPD. Although comorbidity of BPD with other disorders (such as mood, anxiety, and substance abuse disorders) has been assessed before (6), our findings indicate that the clinical impact of comorbid psychotic disorders is much more relevant for the outcome of BPD. According to our analysis, this group of disorders is associated with a poor outcome of BPD and is a predictor for poor outcome; however, for this finding, no comparison can be made due to lack of prior studies in this specific area. What is known, however, is that the presence of auditory verbal hallucinations in BPD is associated with a marked increase of suicidal plans and attempts and with more frequent hospitalization (16). Moreover, it is known that auditory verbal hallucinations in patients with BPD often have a relatively long duration (36, 37) and that their phenomenological characteristics (as well as the distress they evoke) are similar to those in patients diagnosed with schizophrenia (12, 36, 38).

In addition, we found that mood disorders, eating disorders, and somatoform disorders are predictors for a poor outcome of BPD. This is at odds with prior studies, the majority of which reported that comorbid disorders do not predict the outcome of BPD at all (19, 39–42). Incidentally, comorbidity with somatoform disorders was not assessed in these studies.

Thus, our findings for comorbidity with mood and eating disorders as predictors for a poor outcome of BPD are at odds with the majority of studies, whereas no comparison could be made with previous studies on the role of comorbid psychotic disorders and somatoform disorders. This is probably because previous studies did not include patients with more severe and/or complex personality disorders. Another reason might be that most earlier studies focused on lifetime prevalence rates for comorbid disorders, thereby yielding different results.

We do not think that the levels of comorbidity are the result of the specialized nature of the clinic, as our clinic is specialized in the treatment of personality disorders in general and offers no treatment specifically aimed at patients with BPD and psychotic features.

Our finding that also the severity of BPD is a predictive factor for outcome is in line with others (39, 43). Our final finding, that a greater number of years of education is a predictive factor for poor outcome, was based on a rather small difference in years of education between the two groups, i.e., a mean of 11.1 years for the group with a good outcome and 12.2 years for the group with a poor outcome. This finding is difficult to explain, as higher levels of education are generally viewed as predictors for success in life; therefore, replication of this finding is needed.

### Limitations

The present study has several limitations. First, only female patients with BPD were included in the analysis (since only four men had enrolled in the study); therefore, our findings cannot be generalized to men with BPD. Second, because this study had a cross-sectional design, no causal conclusions can be drawn. Third, the sample size of 84 patients was relatively small and may, therefore, have influenced the power of our study. Fourth, because we were unable to collect individual data on the type/types of psychotherapy that the patients had received, no distinction could be made regarding the influence of different forms of psychotherapy on outcome. Fifth, the MINI Neuropsychiatric Interview only assesses the presence of auditory and visual hallucinations and not other hallucinations such as tactile, olfactory, or gustatory hallucinations; the prevalence of psychotic disorder NOS might be higher if these hallucinations were included. Sixth, we did not explore the presence of schizotypal personality disorder. Seven percent of the patients with BPD also fulfill the criteria for a comorbid schizotypal personality disorder (8), which can also be accompanied by psychotic features. The aim of our study, however, was to explore the presence of psychotic disorders in a sample of patients with BPD as a primary diagnosis. We therefore recommend to direct future research at the additional value of schizotypal personality disorder within a population of patients with BPD and comorbid psychotic features. Finally, we did not assess the *severity* of psychiatric comorbidity, which has been associated with greater or lesser symptom improvement (39).

In addition, something that might be construed as a limitation (although in our opinion it is not) is the basic premise of our study, i.e., that BPD and psychotic disorders can be diagnosed simultaneously in individual patients, whereas diagnostic guidelines indicate that, in such cases, a diagnosis of psychotic disorders should prevail over BPD. The reason why we do not concur with this approach is that clinical practice indicates that patients can fulfill the diagnostic criteria of both disorders, that both disorders tend to show only few overlapping symptoms, and that both disorders require their own type of psychiatric treatment. That said, we agree that careful diagnosis is of ultimate importance in such cases and that, sometimes, patients may need to be rediagnosed when either of the two disorders is substantially more prominent than the other, or when patients initially diagnosed with BPD go on to develop a major psychotic disorder. Thus, in the present sample, the two patients diagnosed with “comorbid” schizophrenia and the three with schizoaffective disorder, might have to be relabeled at some point in time as suffering from schizophrenia or a related disorder, rather than from BPD. Nevertheless, this does not contradict the finding that, during our study, they *did* fulfill the diagnostic criteria of both disorders, and that the overwhelming majority of patients in our study did *not* fulfill the criteria for a major psychotic disorder.

### Implications for Clinical Practice and Future Research

Our findings imply that more attention should be paid to the presence of psychotic disorders in patients diagnosed with BPD and, if present, efforts should be directed at offering these patients specialized treatment for both disorders since both require different treatments, and outcome depends substantially on the presence or absence of a comorbid psychotic disorders. That said, the inevitable question of what type of treatment should be offered is not an easy one to answer, since little is known about the treatment of psychosis in patients with BPD. In clinical practice, only a minority of BPD patients receive treatment specifically aimed at their psychotic symptoms. This is reflected in our own patient sample, in which only half of those with a comorbid psychotic disorders were treated with antipsychotics. As an alternative, inspiration might be derived from the field of psychotic disorders, where many evidence-based interventions have been developed, including antipsychotics, cognitive-behavioral therapy, and transcranial magnetic stimulation (44, 45); of these three, only the effects of antipsychotics have been studied in patients with BPD *and* cognitive-perceptual symptoms, such as suspiciousness, referential thinking, paranoid ideation, derealization, depersonalization, illusions, and related perceptual symptoms (46). Three meta-analyses have shown small to moderate effect sizes of this approach (46–48). The reason why effect sizes were not larger might be due to the tendency of clinicians to prescribe relatively low doses of antipsychotics for patients with BPD. In the studies mentioned, the effects of antipsychotics on the severity of specific psychotic symptoms were not assessed. Taken cumulatively, these results indicate that there is a need for further studies on the efficacy of antipsychotics and other interventions for psychotic symptoms and disorders in patients with BPD, preferentially of a design that makes it possible to take the severity of symptoms into account. Regarding psychotherapeutic treatment for BPD with comorbid depression, a number of studies has shown mixed results (49–51), whereas a combined treatment for BPD and eating disorders yielded positive results (52, 53). So far, it is unknown whether other comorbid disorders improve during treatment of BPD, with or without adjuvant strategies specifically directed at them. Therefore, future studies should focus on these types of treatment in BPD with comorbid disorders.

## Conclusion

In our sample, psychotic disorders, especially of the subtype of psychotic disorder NOS, affected 38% of the patients diagnosed with BPD. The presence of such comorbid disorders is associated with a poor outcome and even serves as a predictive factor for poor outcome, along with current mood, eating disorders, and somatoform disorders, the severity of BPD, and more years of education. These findings imply that more attention should be paid to the treatment of psychotic disorders and other comorbid disorders in patients with BPD.

## Ethics Statement

The Stichting Medisch-Ethische Toetsingscommissie Instellingen Geestelijke Gezondheidszorg approved the protocol of this study, study number 6237, CCMO registration number NL1371209706, conform the Guidelines of Helsinki. The procedure was as follows, all documents were presented to this board, during a meeting all documents were discussed, and questions were formulated for the researchers. After the researchers had answered the questions, approval was given to conduct this study.

## Author Contributions

CS and MN contributed to the conception and design of the work and to the acquisition, analysis, and interpretation of data for the work, drafted and revised the work, gave final approval for the final version to be published, and agreed to be accountable for all aspects of the work in ensuring that questions related to the accuracy or integrity of any part of the work are appropriately investigated and resolved. JB and IS contributed to the conception and design of the work and to the analysis and interpretation of data for the work, revised the work, gave final approval for the final version to be published, and agreed to be accountable for all aspects of the work in ensuring that questions related to the accuracy or integrity of any part of the work are appropriately investigated and resolved. MD contributed to the analysis and interpretation of data for the work, revised the work, gave final approval for the final version to be published, and agreed to be accountable for all aspects of the work in ensuring that questions related to the accuracy or integrity of any part of the work are appropriately investigated and resolved.

## Conflict of Interest Statement

The authors declare that the research was conducted in the absence of any commercial or financial relationships that could be construed as a potential conflict of interest.
